# The University of Maryland, Dental Department

**Published:** 1886-10

**Authors:** 


### Editorial, Etc.

---

THE UNIVERSITY OF MARYLAND.—The Dental Department of this old and reputable University began its regular session of 1886 87 with the largest dental class that has ever been South of the Delaware river. And for the first time in the history of dental schools in the Southern section of our country a class is present at the University of Maryland Dental Department numbering considerably over one hundred students.

Quite a number of the present class have passed one session at other dental schools--coming to this University for graduation.

Quite a large number of foreign students are members of the present class, Germany being largely represented, also Canada, S. America and Turkey, the latter country sending two students.

Of the United States nearly every one is represented, Penna., New York and South Carolina leading in number. The Medical Department of the University has also an increased number of students, so that affairs about this old institution present a very busy appearance. The practice in the Dental Infirmary has grown so rapidly as to furnish abundant material for practical work in both branches of dentistry to the large class in attendance. From the fact that the number of dental schools throughout this country has increased so rapidly, the extremely large class at the University of Maryland Dental Department is rather surprising, and can only be accounted for by the wide spread reputation which this dental school has acquired throughout the civilized world.

